# The oncogene Mct-1 promotes progression of hepatocellular carcinoma via enhancement of Yap-mediated cell proliferation

**DOI:** 10.1038/s41420-021-00413-3

**Published:** 2021-03-22

**Authors:** Wenjie Yang, Yong Ni, Shikun Yang, Yang Ji, Xinchen Yang, Feng Cheng, Xuehao Wang, Feng Zhang, Jianhua Rao

**Affiliations:** 1 Hepatobiliary Center, the First Affiliated Hospital of Nanjing Medical University, Nanjing, 210029 China; 2 Department of Hepatopancreatobiliary Surgery, Shenzhen Second People’s Hospital, The First Affiliated Hospital of Shenzhen University, Shenzhen, 518035 China

**Keywords:** Oncogenes, Cancer therapy

## Abstract

Malignant T-cell-amplified sequence 1 (Mct-1) has been reported as an oncogene in multiple malignant diseases. However, the function of Mct-1 in hepatocellular carcinoma (HCC) and the molecular mechanisms underlying tumor progression have not been explored. In this study, Mct-1 expression levels in HCC tissues and cells were detected by quantitative real-time PCR and western blotting. Mct-1 shRNAs and overexpression vector were transfected into HCC cells to downregulate or upregulate Mct-1 expression. In vitro and in vivo assays were performed to investigate the function of Mct-1 in cell proliferation and apoptosis. RNA sequencing analysis (RNA-seq) was performed to explore differences in gene expression when silenced Mct-1 expression. Mct-1 was upregulated in HCC specimens and cell lines, and higher expression of Mct-1 was predictive of poor survival. Overexpression of Mct-1 was shown to promote cell proliferation and repress cell apoptosis both in vitro and in vivo. The results of RNA-seq indicated that knockdown of Mct-1 suppressed Yap expression, while the results of the luciferase assay also revealed that Mct-1 increases the activity of the Yap promoter. Restoration of Yap expression in Mct-1 knockdown cells partially recovered the promotion of cell proliferation and inhibition of apoptosis. Collectively, these results indicate that Mct-1 acts as a tumor promoter gene in HCC progression by up-regulating Yap expression and, thus, could serve a novel potential diagnostic and prognostic biomarker for HCC.

## Introduction

Liver cancer was the sixth most common cancer and the fourth leading cause of cancer-related death worldwide^1,2^. Hepatocellular carcinoma (HCC) is the most common form of primary liver cancer, accounting for 75–85% of cases, and shares the similar characteristic of chronic liver inflammation as with long-term infection of hepatitis viruses B and C, nonalcoholic fatty liver disease, and alcohol abuse. Although considerable progress has been made in the diagnosis and treatment of HCC, the overall 5-year survival rate remains low as a result of tumor recurrence and metastasis^3–5^. Hence, there is an urgent need to elucidate the molecular mechanisms underlying the progression of HCC in order to advance treatment strategies.

Cell autonomous and non-autonomous mechanisms collectively determine tumor development and progression. Malignant T-cell-amplified sequence 1 (Mct-1), which was first identified as a putative oncogene in human T-cell lymphoma and localized to the long arm of chromosome Xq22-24, codes for an oncogenic protein that promotes the development of human malignant lymphoma^6^. Recent studies have reported crucial functions of Mct-1 in multiple cancer biological processes, including cell proliferation, the cell cycle, cancer stemness, and the intracellular generation of reactive oxygen species and mitochondrial superoxide^7–9^. In addition, mounting evidence has revealed that Mct-1 can specifically combine with and regulate the viability of the p53 gene promoter and p53 mRNA stability in breast cancer and non-small cell lung cancer. Mct-1 overexpression accompanied by p53 deficiency synergistically promotes chromosome instability and tumor survival, suggesting that Mct-1 can potentially function as a transcriptional regulatory factor to regulate tumor progression^10–12^. However, the actual function of Mct-1 in HCC remains unclear.

The Hippo signaling pathway is a recently identified tumor suppressor signaling pathway that participates in the regulation of organ size and cell proliferation. Transcription factor Yes-associated protein (Yap) is the core downstream effector of this pathway^11,13,14^. Under a normal physiological state, Hippo kinase (Mst1/2) phosphorylates downstream Last1/2 kinase and phosphorylated Last1/2 can further promote the phosphorylation of Yap. In mammals, Mst1 is the analogue of Hippo and is widely expressed in vivo. Phosphorylated Yap is usually trapped and degraded in the cytoplasm via the proteasome pathway. However, this signaling pathway is dramatically inhibited in cancer cells. Unphosphorylated Yap is translocated into the nucleus, where it promotes cell proliferation, stemness, and invasion capabilities. Multiple studies have demonstrated that genetic inhibition of Mst1 or aberrant Yap expression leads to the spontaneous formation of tumor-initiating cells and the progression of HCC^15–17^. However, the precise relationship between Mct-1 and the Hippo signaling pathway has not yet been investigated.

In this study, Mct-1 was dramatically upregulated in the majority of HCC cell lines and 112 HCC tumor specimens, as compared with normal liver cells and adjacent tissues. In addition, Mct-1 promoted the proliferation and inhibited apoptosis of HCC cells both in vitro and in vivo. Furthermore, the results demonstrated that Mct-1 enhanced the activity of the Yap gene promoter. Inhibition of Yap expression in cells overexpressing Mct-1 partially arrested the pro-tumorigenic effect of Mct-1. Taken together, these results highlight the indispensable role of Mct-1 in the progression of HCC, suggesting that Mct-1 may be an effective therapeutic target against HCC.

## Results

### Mct-1 expression is significantly upregulated in HCC patients and cell lines

**Fig. 1:**
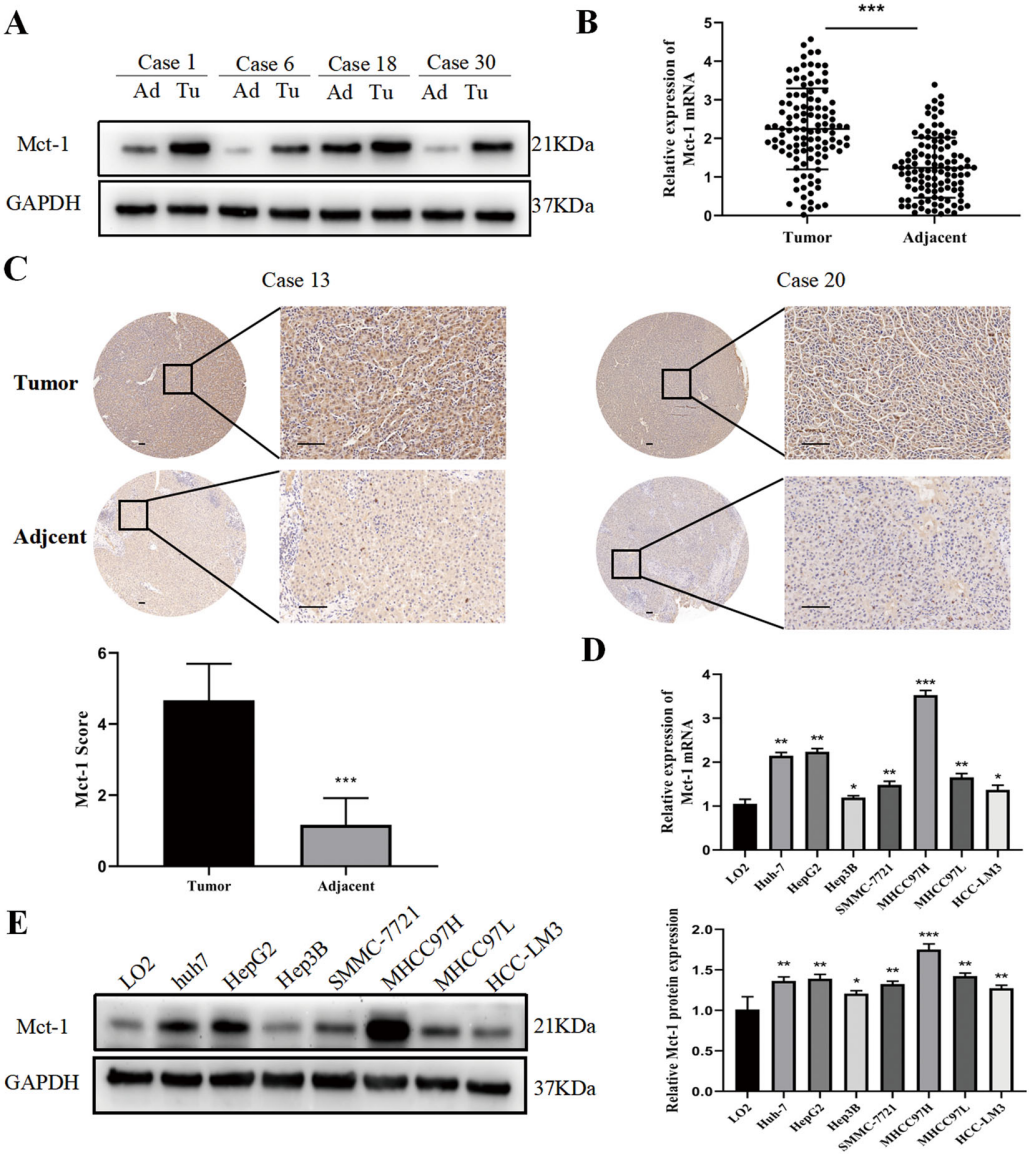
Mct-1 expression is significantly upregulated in HCC tissues and cell lines. Mct-1 protein (**A**) and mRNA (**B**) levels in HCC tumor tissues and corresponding adjacent nontumor tissues. **C** Representative IHC staining of Mct-1 in HCC tumor tissues as compared with peritumor tissues. Scale bar = 50 µm. **D** Mct-1 mRNA and **E** protein expression levels in HCC cell lines and normal LO2 cells by qRT-PCR and western blot analyses. *p < 0.05, **p < 0.01, ***p < 0.001.

In order to analyze the role of Mct-1 in the progression of HCC, Mct-1 expression at the protein and mRNA levels was measured in 112 surgically resected tumor tissues and matched non-tumor or normal liver tissues from HCC patients. The results showed that Mct-1 protein and mRNA levels were higher in tumor tissues than matched adjacent normal tissues (Fig. 1A, B). Data regarding Mct-1 expression in HCC tissues were retrieved from The Cancer Genome Atlas database as references. Significantly greater Mct-1 expression was confirmed in 371 HCC tissues samples as compared to adjacent normal tissues (Fig. S1). To further investigate the value of Mct-1 as a marker of the progression of HCC, IHC staining of Mct-1 protein expression in HCC and adjacent normal liver tissues was conducted, which demonstrated that Mct-1 staining was markedly increased in HCC tissues (Fig. 1C).

In addition, Mct-1 expression was significantly increased HCC cells lines as compared to normal LO2 liver cells. The results further showed that Mct-1 expression at the mRNA and protein levels was lower in normal LO2 cells and immortalized Hep3B cells, while highly expressed in MHCC97H cells (Fig. 1D, E). Thus, Hep3B and MHCC97H cells were chosen for subsequent experiments. Together, these results revealed that Mct-1 expression was significantly increased in HCC tissues and cell lines.

### Upregulated Mct-1 expression is predictive of a poor prognosis

**Fig. 2:**
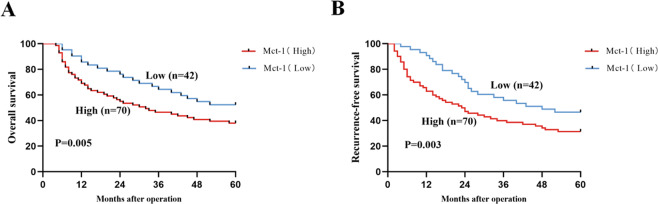
Upregulated Mct-1 expression is predictive of a poor prognosis of HCC patients. Kaplan–Meier curves of OS (**A**) and RFS (**B**) of HCC patients based on Mct-1 expression levels.

To explore the clinical significance of Mct-1 expression in HCC, tissue specimens from 112 patients were assigned to a high or low Mct-1 expression group for analysis of possible correlations between clinicopathological features and the prognosis of HCC patients (Table 1). The results demonstrated positive correlations between Mct-1 levels and tumor size (*p* = 0.011), tumor multiplicity (*p* = 0.029), and tumor-node-metastasis (TNM) stage (*p* = 0.036), which indicated an important role of Mct-1 in the regulation of HCC progression. Further analysis revealed that Mct-1 expression was inversely associated with the OS and RFS rates of HCC patients (*p* = 0.005, Fig. 2A; *p* = 0.003, Fig. 2B). In other words, lower Mct-1 expression was associated with better OS and RFS rates. These data indicate that Mct-1 is a potential novel indicator of HCC prognosis and latent progression.

**Table 1:** Association between Mct-1 expression and clinicopathologic features of patients with HCC (*n* = 112).

Clinicopathological indexes	Mct-1 expression		*χ*^2^	*P* value
	High	Low		
All cases	70	42		
Age (years)				
≤60	37	25	0.472	0.492
>60	33	17		
Gender				
Male	41	28	0.727	0.394
Female	29	14		
HBs antigen				
Absent	13	9	0.136	0.713
Present	57	33		
Liver cirrhosis				
With	53	36	1.609	0.205
Without	17	6		
AFP (ng/mL)				
≤20	27	13	0.664	0.415
>20	43	29		
Tumor size				
≤3	23	24	6.357	0.011^a^
>3	47	18		
Edmondson grade				
I–II	32	12	3.234	0.072
III–IV	38	30		
Tumor multiplicity				
Single	39	32	4.743	0.029^a^
Multiple	31	10		
TNM stage				
I–II	29	26	4.404	0.036^a^
III–IV	41	16		

^a^Indicates *p* < 0.05.

### Mct-1 has potent oncogenic functions in HCC

**Fig. 3:**
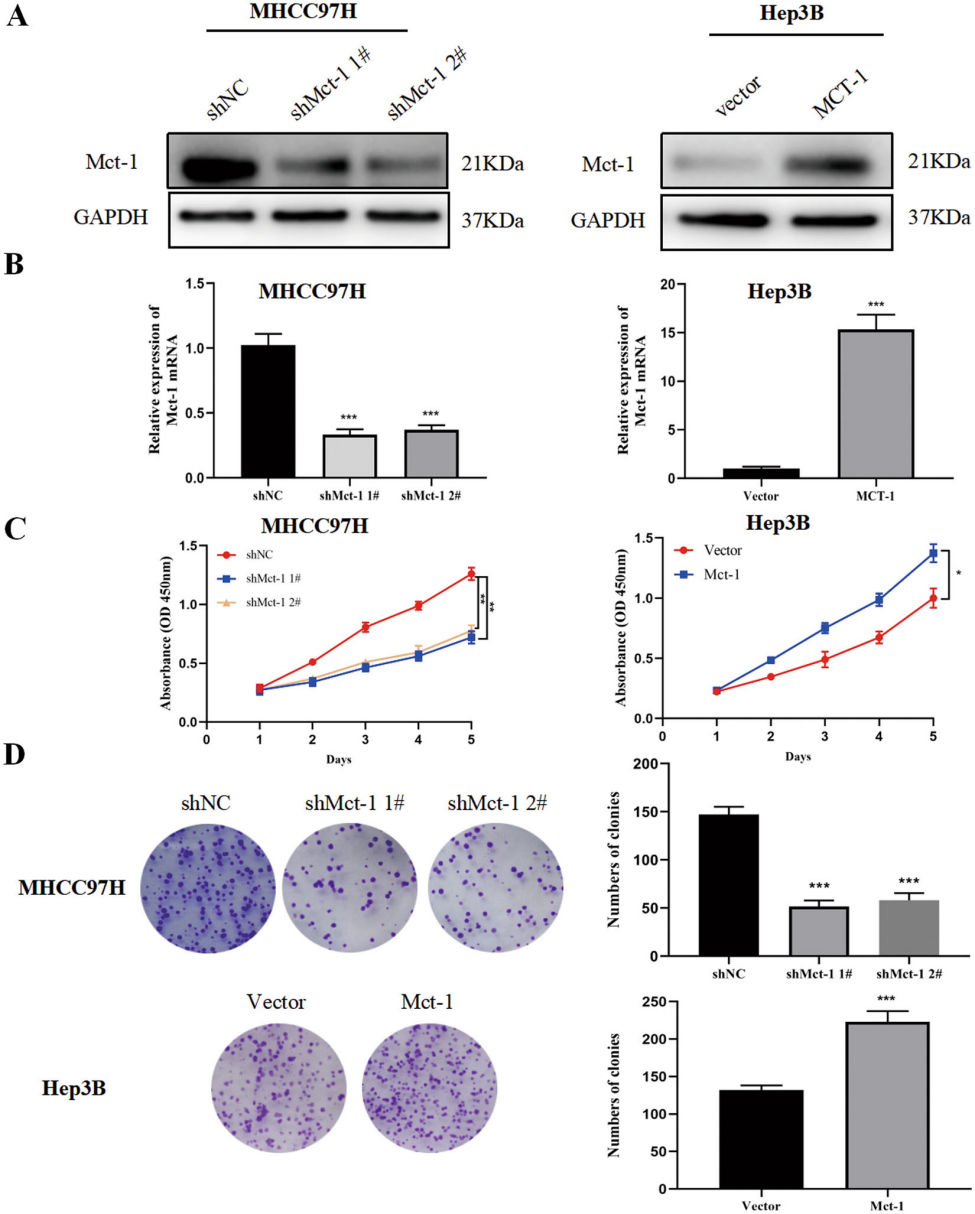
Mct-1 has potent oncogenic functions in HCC. MHCC97H cells transfected with shRNA targeting Mct-1 were defined as shMct-1. Cells transfected with empty lentiviral vectors were established as negative controls (shNC). Hep3B cells were transfected with lentiviruses overexpressing Mct-1 and defined as Mct-1. Cells transfected with empty lentiviral vectors were used as controls (Vector). Mct-1 expression was validated by western blot (**A**) and qRT-PCR (**B**) analyses. **C** The CCK-8 assay results of MHCC-97H and Hep3B cells following silencing or overexpression of Mct-1. **D** Colony formation assay results of MHCC-97H and Hep3B cells after knockdown or overexpression of Mct-1. The data are presented as the mean ± standard deviation (SD) of experiments performed in triplicate. *p < 0.05, **p < 0.01, ***p < 0.001.

Previous studies have reported that overexpression of Mct-1 promotes the proliferation and progression of multiple invasive cancers^18,19^. However, the potential function of Mct-1 in HCC remains unclear. To assess the role of Mct-1 in the tumorigenesis of HCC, lentivirus-mediated Mct-1-knockdown MHCC97H cells and Mct-1-overexpressing Hep3B cells were established. Western blot and qRT-PCR analyses were used to evaluate transfection efficiency. As shown in Fig. 3A, B, Mct-1 expression levels were significantly repressed in MHCC97H cells, while dramatically increased in Hep3B cells.

**Fig. 4:**
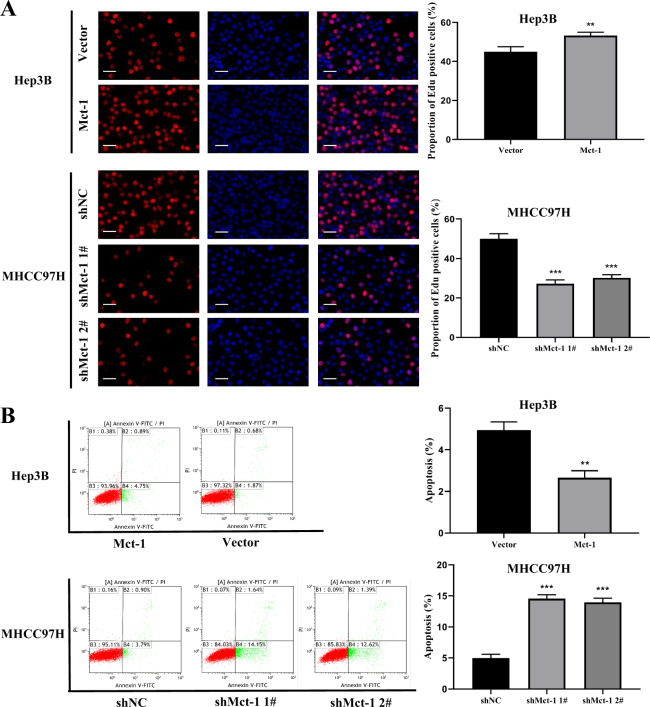
Mct-1 has potent oncogenic functions in HCC. **A** The Edu assay was used to assess the proliferation of MHCC-97H and Hep3B cells following Mct-1 knockdown or overexpression. Scale bar = 50 µm. **B** Flow cytometry was performed to assess the apoptosis rate of MHCC-97H and Hep3B cells following Mct-1 knockdown or overexpression. The data are presented as the mean ± SD of experiments performed in triplicate. *p < 0.05, **p < 0.01, ***p < 0.001.

The CCK-8, colony formation, and Edu assays were performed to investigate the role of Mct-1 in the proliferation of HCC cells. The results showed that proliferation was markedly boosted in cells overexpressing Mct-1, while Mct-1 knockdown significantly suppressed this trend. The results of the CCK-8 assay showed that Mct-1 overexpression increased the viability of Hep3B cells, whereas Mct-1 inhibition suppressed the viability of MHCC97H cells (Fig. 3C). Meanwhile, aberrant Mct-1 expression enhanced the colony formation capabilities of Hep3B cells, while Mct-1 knockdown inhibited the growth of MHCC97H cells (Fig. 3D). The results of the Edu assays further confirmed the influence of Mct-1 on the proliferation of HCC cells (Fig. 4A). These results showed that increased Mct-1 expression significantly elevated the proliferation of Hep3B cells, while knockdown of Mct-1 had an opposite effect in MHCC97H cells.

Cell apoptosis is the key factor in tumor formation and progression. Thus, flow cytometry was employed to evaluate the impact of Mct-1 on cell apoptosis. The results showed that the proportion of apoptotic cells was greater in the Mct-1-knockdown MHCC97H group than the shNC group, while overexpression of Mct-1 significantly decreased the apoptosis rate (Fig. 4B). These results revealed that Mct-1 promotes the proliferation of HCC cells and inhibits cell apoptosis in vitro.

### Yap expression is closely related to Mct-1 expression in vitro

**Fig. 5:**
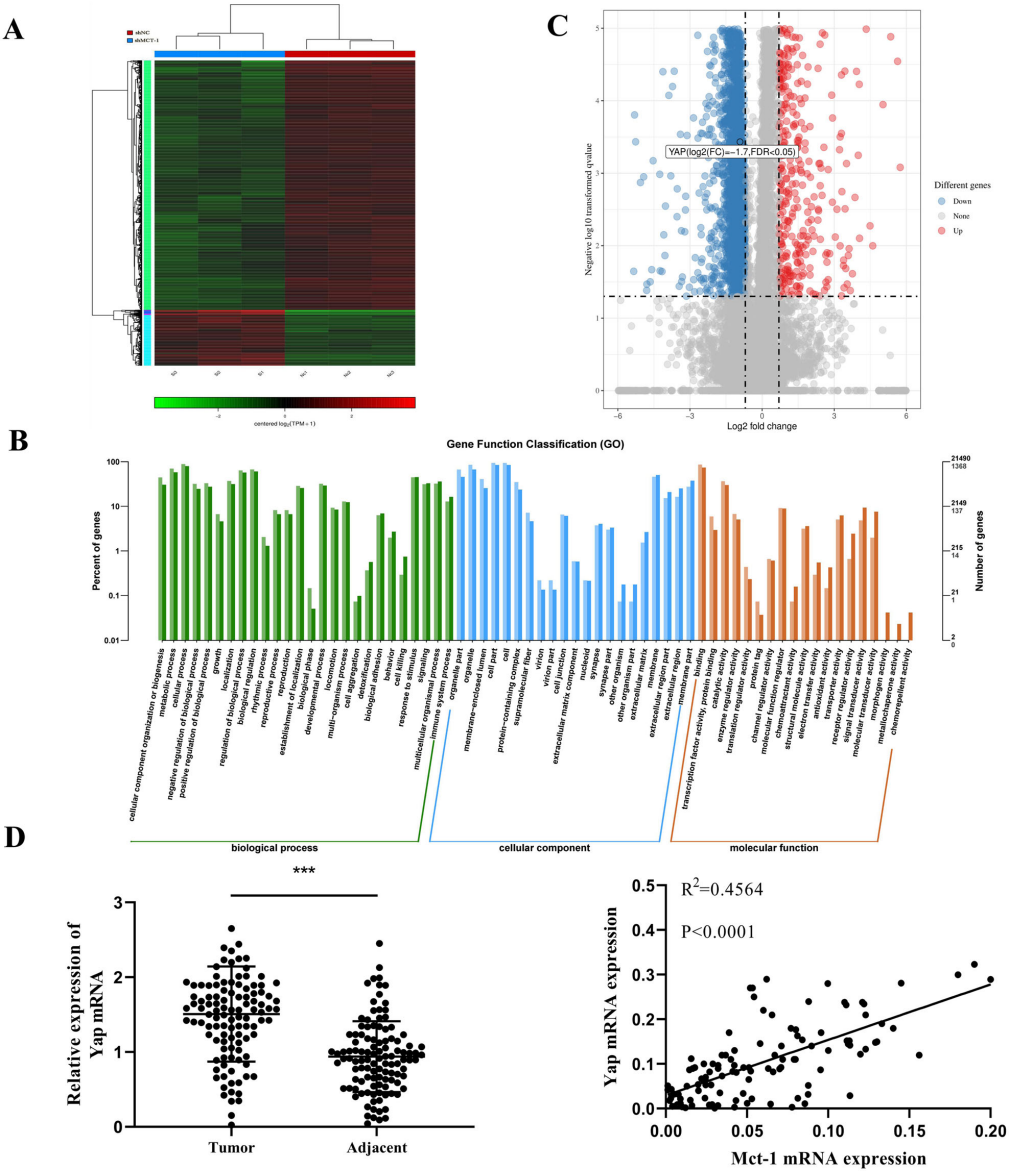
Yap expression is closely related to Mct-1 expression in vitro. **A** A heatmap of DEGs in the Mct-1 silenced (shMct-1) and negative control (shNC) groups. DEGs with a log^2^ fold change (FC) > 1 are indicated in red, while those with a log^2^ FC < 1 are indicated in green. **B** Gene function classification analysis of the shMct-1 and shNC groups of MHCC97H cells. **C** A volcano plot of DEGs in the shMct-1 and shNC groups of MHCC97H cells. DEGs with a log^2^ FC > 1 are indicated in red, while those with a log^2^ FC < 1 are indicated in blue. **D** qRT-PCR was used to examine the expression of Yap in 112 pairs of HCC tissues and corresponding peritumor tissues. **E** The expression patterns of Mct-1 and Yap were positively correlated in HCC specimens. The data are presented as the mean ± SD of experiments performed in triplicate. *p < 0.05, **p < 0.01, ***p < 0.001.

To explore the potential mechanisms of Mct-1 in cell proliferation and apoptosis, Mct-1-silenced MHCC97H cells and control cells we selected for RNA-seq analysis to identify differences in gene expression patterns. In this study, 310 differentially expressed genes (DEGs) were upregulated, while 1389 DEGs were downregulated following Mct-1 silencing (Fig. 5A). Gene ontology analysis implicated the involvement of Mct-1 in transcription factor activity, molecular function regulator, and regulation of the cellular biological process, suggesting that Mct-1 may regulate cancer-related signaling pathways through transcriptional regulation (Fig. 5B).

It is well accepted that the repression of the Hippo signaling pathway can induce uncontrolled cell proliferation and the occurrence of HCC. The oncogene Yap, as the major downstream effector of this signaling pathway, plays fundamental roles in the regulation of cell proliferation and apoptosis. However, it remains unclear whether there is a correlation between Mct-1 and Yap expression. The results of RNA-seq analysis suggest that Mct-1 markedly reduced Yap expression (Fig. 5C).

To better understand the correlation between Mct-1 and Yap, qRT-PCR was performed to explore the expression patterns of Yap in HCC specimens. The results indicated that Yap expression was greater in the tumor specimens than the adjacent tissues. The results of correlation analysis indicated positive correlations between the expression patterns of Mct-1 and Yap in HCC (Fig. 5D, E).

### Mct-1 increase Yap expression through its transcription factor activity

**Fig. 6:**
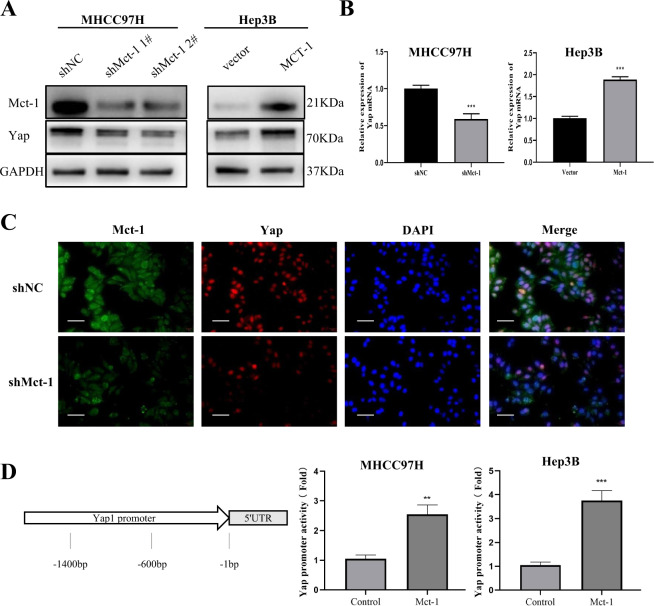
Mct-1 increases Yap expression through its transcription factor activity. Yap expression was assessed by western blot (**A**) and qRT-PCR (**B**) analyses. **C** Immunofluorescent staining images of Mct-1 and Yap in MHCC97H-NC and MHCC-97H-shMct-1. Scale bar = 50 µm. **D** Primers specific for the Yap promoter region (−1400 to −1) were designed for the dual-luciferase reporter assay. The luciferase activity of the pGL3-Yap-Luc vector was enhanced in MHCC97H and Hep3B cells after transfection with the Mct-1 plasmid. The data are presented as the mean ± SD of experiments performed in triplicate. *p < 0.05, **p < 0.01, ***p < 0.001.

Next, Yap expression in cells following knockdown or overexpression of Mct-1 was compared with that of the control cells. The results of western blot analysis showed that Yap expression was downregulated in Mct-1-knockdown MHCC97H cells as compared with the control group, but significantly upregulated in Mct-1-overexpressing Hep3B cells (Fig. 6A, B). In addition, immunofluorescence also confirmed a consistent trend between the expression profiles of Mct-1 and Yap (Fig. 6C).

Furthermore, to investigate whether Mct-1 influences intrinsic Yap gene activation, MHCC97H and Hep3B cells were transfected with a pcDNA vector alone or pcDNA-Mct-1. To examine the function of the Yap promoter, a 1.4-kb Yap gene promoter segment (−1400 to −1) was engineered into a pGL3-luciferase basic vector (pGL3-Yap-Luc), which was introduced into MHCC97H and Hep3B cells that expressed the vector alone (pcDNA) or a vector encoding Mct-1 (pcDNA-Mct-1). The results showed that Mct-1 promoted activation of the Yap promoter in both cell lines, especially in Hep3B cells (Fig. 6D). Together, these results suggest that Mct-1 positively regulates Yap expression through its transcription factor activity.

### Yap expression is critical for Mct-1-mediated promotion of cell proliferation and inhibition of cell apoptosis

**Fig. 7:**
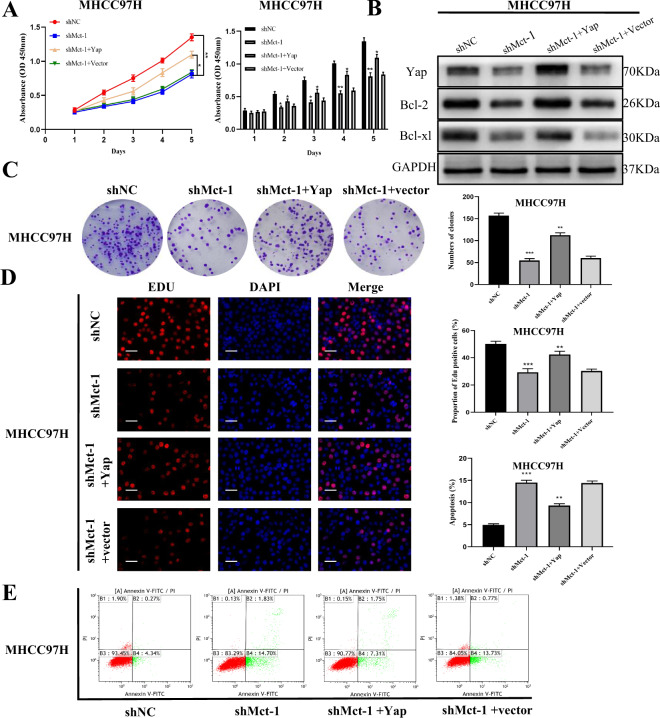
Yap expression is critical for Mct-1-mediated promotion of cell proliferation and inhibition of cell apoptosis. **A**–**D** CCK-8, colony formation, Edu, and cell apoptosis analyses of Mct-1-knockdown MHCC97H cells transfected with the Yap plasmid or vector. Scale bar = 50 µm. **E** Protein expression of Yap together with the anti-apoptotic proteins Bcl-2 and Bcl-xl in MHCC97H cells transfected with the Mct-1 knockdown lentivirus (shMct-1) and Yap-overexpression plasmid. The data are presented as the mean ± SD of experiments performed in triplicate. *p < 0.05, **p < 0.01, ***p < 0.001.

To explore whether Yap is involved in the tumor-promoting effects of Mct-1 in HCC, Yap expression was upregulated in Mct-1-knockdown MHCC97H cells using a Yap-overexpression plasmid, while Yap was silenced in Mct-1-overexpressing Hep3B cells using specific siRNA. Yap expression was confirmed by western blot analysis (Fig. 7B). The results of the CCK-8, colony formation, and Edu assays showed that increased Yap expression significantly reversed the cell proliferation inhibition effects in Mct-1-knockdown MHCC97H cells, while silencing Yap repressed the proliferation of Hep3B cells overexpressing Mct-1 (Fig. 7A, C, D; Fig. S2A, C, D). In addition, the results of flow cytometry showed that restoration of Yap expression in Mct-1-knockdown MHCC97H cells decreased the cell apoptosis rate and inhibited expression of the anti-apoptotic proteins Bcl-2 and Bcl-xl (Fig. 7E, B). However, silencing of Yap in Mct-1-overexpressing Hep3B cells had the opposite effect (Fig. S2E, B). Collectively, these results show that Yap is essential for the promotion of Mct-1-induced proliferation and inhibition of cell apoptosis in HCC.

### Mct-1 promotes the progression of HCC in vivo

**Fig. 8:**
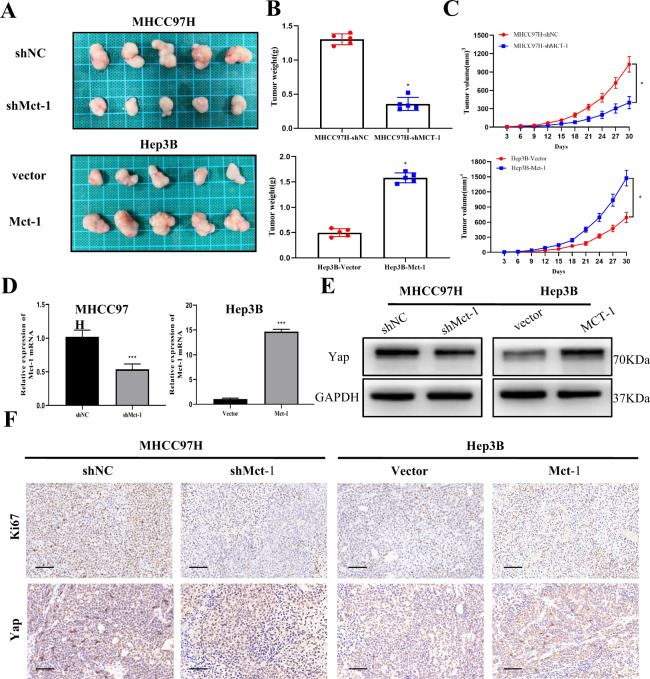
Mct-1 promotes HCC progression in vivo. **A**–**C** Representative photographs of tumors derived from lentivirus-mediated Mct-1-silenced MHCC97H cells and Mct-1-overexpressing Hep3B cells. The tumor volume and weight were measured. **D** The Mct-1 expression levels of the xenografts were analyzed by qRT-PCR. **E** Yap expression levels of xenografts were detected by western blot analysis. **F** Representative immunostaining images of the Ki-67 and Yap expression levels in xenografts. Scale bar = 50 µm. The data are presented as the mean ± SD of experiments performed in triplicate. *p < 0.05, **p < 0.01, ***p < 0.001.

To further examine the effect of Mct-1 on the progression of HCC in vivo, MHCC97H and Hep3B cells transfected with lentiviruses, as previously described, were subcutaneously injected into nude mice. Tumor growth was recorded every 3 days, and the mice were sacrificed on post-inoculation day 30. The xenografts generated from the Mct-1-overexpressing Hep3B group grew faster and were more sustainable than those from the empty vector group. However, Mct-1 knockdown obviously suppressed the tumor growth rate in the Mct-1-knockdown MHCC97H group as compared with the negative control group (Fig. 8A–C). The results of qRT-PCR showed higher Mct-1 expression levels in the Hep3B cells transfected with Mct-1 than the empty vector group, whereas expression was lower in MHCC97H cells transfected with the Mct-1 inhibitor. In addition, the results of western blot analysis showed that Yap protein expression was decreased in the Mct-1-knockdown MHCC97H cells as compared with the negative control group, but increased in the Mct-1-overexpressing Hep3B cells (Fig. 8D, E).

Furthermore, the xenografts were stained for Ki-67 and Yap. The results revealed that Mct-1 plays a vital role in the proliferation of HCC cells and there is a positive correlation between the expression patterns of Mct-1 and Yap (Fig. 8F). Taken together, these data indicate that Mct-1 promotes HCC tumorigenesis and enhances Yap expression in vivo.

## Discussion

According to current guidelines, surgical resection is the first-line option for liver tumor patients. However, the majority of patients are diagnosed in the advanced stage of the disease, thus surgery is no longer an option for these patients due to delayed diagnosis and metastasis. Furthermore, HCC is not sensitive to other therapeutic interventions, such as radiotherapy and chemotherapy^20–22^. Even though the application of targeted drugs provides new strategies for the treatment of HCC, only 30% of patients benefit from such regimens because of severe adverse side effects and acquired resistance^23,24^. Therefore, it is necessary to development new and effective regimens for the treatment for HCC.

Mct-1 reportedly plays a prominent role in the regulation of cell growth and proliferation^25^. In this study, Mct-1 was remarkably upregulated in HCC tissues as compared with adjacent tissues. In addition, Mct-1 was identified as an independent risk factor for postoperative recurrence and OS in patients with HCC. High expression of Mct-1 is predictive of poor 5-year OS and shorter RFS, and closely correlated with poor prognostic characteristics, such as tumor size, TNM stage, and tumor histological classification as compared with low Mct-1 expression. In addition, the results of the present study demonstrated that Mct-1 promotes the proliferation of tumor cells and inhibited apoptosis both in vitro and in vivo, indicating that Mct-1 upregulation is involved in the progression of HCC. To explore the molecular mechanisms underlying tumor progression mediated by Mct-1, the results of RNA-seq analysis suggested that knockdown of Mct-1 repressed Yap expression.

Yap, as the key effector of the Hippo signaling pathway, is crucial for organ growth and cell proliferation. Overwhelming evidence indicates that Yap promotes cell proliferation and enhances the self-renewal capability of cancer stem cells. In addition, Yap is reported to repress the expression of tumor suppressor factor p53, via enhancing the transcriptional activity of mutant p53^26–28^. The results of the present study revealed a positive correlation between the expression profiles of Mct-1 and Yap. Silencing of Yap reversed the proliferation and anti-apoptosis capabilities of cells overexpressing Mct-1. Furthermore, the results of the luciferase activity assays illustrated that Mct-1 regulates the activation of the Yap promoter. A previous study revealed that overexpression of Mct-1 promoted the expression of Yin Yang 1 (YY1) and epidermal growth factor receptor. Notably, Yap exerts a synergistic effect through the recruitment of YY1 and enhancer of zeste homologue 2 (EZH2), which plays a key role in regulating the cyclin-dependent kinase inhibitor p27 and overriding contact inhibition between cells. Further investigations revealed that both YY1 and EZH2 are necessary for Yap-mediated repression of p27 expression^29,30^. These results unveiled that Mct-1 has the potential to promote HCC progression via the regulation of p27 expression and impairing the effect of contact inhibition in HCC. Hence, regulation of Mct-1 expression may be beneficial to inhibit the progression of HCC.

However, it remains unclear whether there are other specific mechanisms, such as the protease degradation pathway or post-transcriptional control, involved in the regulation of Mct-1 and Yap expression. For instance, Mct-1 forms a dimeric complex with density-regulated protein and analogous to eukaryotic translation initiation factor 2D, which is closely involved in the process of tRNA recruitment and translation re-initiation in various human diseases^31^. Interestingly, a recent study also indicated that Mct-1 activation facilitates the release of multiple cytokines, such as interleukin-6, monocyte chemotactic protein 1, and granulocyte-macrophage colony-stimulating factor via interacting with miR-34a, and is also highly correlated to the recruitment of tumor-associated macrophages (TAMs), especially accumulation of the tumor-promoting M2 type^32^. These results indicate that Mct-1 is also involved in regulating the recruitment and polarization of TAMs. Thus, further studies are warranted to determine if Mct-1 plays a role in the regulation of the tumor micro-environment.

In conclusion, the results of the present study revealed that Mct-1 is associated with poor prognosis and promotes the progression of HCC both in vitro and in vivo via enhancing Yap-mediated cell proliferation and apoptosis inhibition. To the best of our knowledge, this is the first report of Mct-1 as a promoter in HCC. These findings provide a new option for targeted molecular therapies for HCC.

## Methods

### Study approval

The study protocol was approved by the Research Ethics Committee of the First Affiliated Hospital of Nanjing Medical University (Nanjing, China) and conducted in accordance with the ethical guidelines outlined in the Declaration of Helsinki. Informed consent forms were obtained from all patients or their relatives for the use of human tissue samples.

### Clinical specimens

A total of 112 HCC specimens were obtained from 120 patients undergoing primary HCC hepatectomy at the First Affiliated Hospital of Nanjing Medical University between 2017 and 2019. Patients who met the following criteria were considered for study inclusion: (1) initial diagnosis of HCC and no preoperative treatment; (2) no distant metastases; and (3) curative liver resection. Small sections of liver cancer tissues and adjacent tissues (>5 cm from the edge of the cancer tissue) were collected, immediately frozen, and embedded in paraffin at the Department of Pathology.

### Cell culture

Immortalized human hepatocyte LO2 cells and the human HCC cell lines Huh7, MHCC97H, MHCC97L, SMMC-7721, HepG2, Hep3B, and HCC-LM3 were purchased from the American Type Culture Collection (Manassas, VA, USA) and cultured in Dulbecco’s modified Eagle’s medium (Life Technologies, Carlsbad, CA, USA) supplemented with 10% fetal bovine serum (Life Technologies) and 100 μg/mL of penicillin/streptomycin (Invitrogen Corporation, Carlsbad, CA, USA) at 37 °C under an atmosphere of 5% CO_2_/95% air.

### Western blot analysis

Western blot analysis was performed as described previously^33^ with primary antibodies against Mct-1 (ab156807, ab238825; Abcam, Cambridge, UK), YAP (ab52771; Abcam), Bcl-2 (ab32124; Abcam), Bcl-XL (ab32370; Abcam), and glyceraldehyde 3-phosphate dehydrogenase (GAPDH; ab8245; Abcam);

### Quantitative reverse transcription-polymerase chain reaction (qRT-PCR)

Total RNA was extracted from HCC tissues and cells with the use of TRIzol reagent (Invitrogen) and reverse-transcribed into complementary DNA (cDNA) with a PrimeScript RT Reagent kit (Vazyme Biotech Co., Ltd., Nanjing, China) in accordance with the manufacturers’ instructions. The primers for amplification of Mct-1 and Yap were designed by Guangzhou RiboBio Co., Ltd. (Guangzhou, China). qRT-PCR was performed using ChamQ Universal SYBR qPCR Master Mix (Vazyme) and a 7900 Real-time PCR system (Applied Biosystems, Carlsbad, CA, USA). GAPDH was selected as an internal control. The results were calculated using the 2^−ΔΔCT^ method. The following primers were used for amplification of the target mRNA and internal control: Mct-1 forward (5′-CTG CAT CCA GTT GAA AAC TTC AG-3′) and reverse (5′-GGC ATC GGA CTA TTT TGA CAG G-3′); Yap forward (5′-AGG AGA CAC ATG CAC CGG A-3′) and reverse: 5′-CAG CAG CAA TGG ACA AGG AA-3′); and GAPDH forward (5′-GGA GCG AGA TCC CTC CAA AAT-3′) and reverse (5′-GGC TGT TGT CAT ACT TCT CAT GG-3′).

### RNA sequencing

RNA was extracted from Mct-1 knockdown or control type MHCC97H cells using TRIzol Reagent (Invitrogen) according to the manufacturer’s protocol. Polyadenylated RNA was isolated from 5 μg total RNA with the mRNA Capture Beads. RNA samples were analyzed by RNA sequencing (AOYIN MEDICAL TECHNOLOGY, JiangSu, China) based on the manufacturer’s protocols. Illumina Hiseq™ platform was used to sequence the samples for the subsequent generation of raw data. Genes significantly differentially expressed between sh-NC and sh-Mct-1 cells were selected based on fold change ≥1.0 and *P* ≤ 0.05 using the DEGseq method.

### Immunohistochemical (IHC) analysis

Tissues that were fixed in 4% paraformaldehyde and embedded in paraffin were cut into 4-µm-thick slices, which were mounted on slides, heated with 10 mmol/L sodium citrate buffer (pH 6.0) for antigen repair, blocked with 5% goat serum for 10 min, and incubated with primary antibodies against Mct-1, Yap, or Ki67 overnight at 4 °C. The next day, the specimens were incubated with secondary antibodies at 37 °C for 30 min, treated with 3,3ʹ-diaminobenzidine for 5 min, and stained with hematoxylin. The extent of IHC staining was scored as the percentage of positively stained cells (score 0, 0–5%; score 1, 6–35%; score 2, 36–70%; and score 3, >70%) and staining intensity (score 0, negatively stained; score 1, weakly stained; score 2, moderately stained; and score 3, strongly stained). The final score was calculated as the positive cells score × staining intensity score (“0” for a score of 0–1, “1” for a score of 2–3, “2” for a score of 4–6, and “3” for a score of >6). The Mct-1, Yap, and Ki67 expression status were graded by two independent experienced pathologists.

### Immunofluorescence staining

The tumor cells were fixed with pre-cooled formaldehyde for 30 min, infiltrated with 0.1% Triton X-Mel 100 for 15 min, washed twice with phosphate-buffered saline (PBS), blocked with 5% bovine serum albumin for 1 h at room temperature, and then incubated with primary antibodies against Mct-1 or Yap overnight at 4 °C. The next day, the cells were incubated with secondary antibodies at 37 °C for 30 min, re-stained with 4′,6-diamidino-2-phenylindole (DAPI), and observed under a fluorescence microscope (Leica Microsystems GmbH, Wetzlar, Germany).

### Cell transfection

Human HCC MHCC97H and Hep3B cells were transfected with lentiviruses for Mct-1 knockdown, a negative control (shMct-1/shNC), overexpression of Mct-1, or scrambled (Mct-1/Vector) (GenePharma Co., Ltd., Shanghai, China) in accordance with the manufacturer’s protocol. After incubating in a medium containing lentiviral particles at 37 °C for 24 h, stably transfected MHCC97H and Hep3B cells were selected over a period of 14 days with the use of 5 μg/mL of puromycin (Sigma-Aldrich Corporation, St. Louis, MO, USA). All vectors were verified by sequencing.

### Yap knockdown and overexpression

Full-length Yap cDNA was cloned into the pCDNA3.1(+) vector to generate Yap expression vectors. Small interfering RNA (siRNA) specific for Yap and a nonspecific duplex oligonucleotide as negative control were synthesized by GenePharma Co., Ltd. The cells were transfected with full-length Yap cDNA and siRNA using Lipofectamine 3000 Transfection Reagent (Thermo Fisher Scientific, Waltham, MA, USA) in accordance with the product manual. Yap expression was determined by qRT-PCR and western blot analysis.

### Cell proliferation and colony formation capability

The cell-counting kit 8 (CCK-8) assay was used to evaluate the proliferation of HCC cells as previously reported^34^. For the colony formation assay, 2000 cells were inoculated in a 60-mm dish and incubated for 14 days to promote colony formation. The cell colonies were fixed with 4% paraformaldehyde for 30 min, washed twice with PBS, stained with 0.5% crystal violet (Sigma-Aldrich Corporation) for 15 min, and counted.

### The 5-ethynyl-2′-deoxyuridine (Edu) proliferation assay

Cell proliferation was evaluated using an Edu assay kit (Guangzhou RiboBio Co., Ltd., Guangzhou, China). Briefly, 2 × 10^5^ cells were incubated in the wells of a 12-well plate for 24 h, treated with 2 mg/mL of glycine and 0.5% Triton X-100 for 30 min, and stained with Apollo staining reaction buffer for 30 min. The cell nuclei were stained with DAPI for 15 min. After washing twice with PBS, the Edu incorporation rate was analyzed under a fluorescence microscope.

### Apoptosis analysis

The proportion of apoptotic cells was determined using a FITC Annexin V Apoptosis Detection Kit (Vazyme). Briefly, 5 × 10^5^ cells were collected, washed twice with PBS, resuspended in 300 μL of binding buffer, and stained with 3 μL of Annexin V-phycoerythrin and 3 μL of propidium iodide, respectively. After incubation for 15 min, the stained cells were subjected to flow cytometry using a FACSCalibur™ Flow Cytometer (BD Biosciences, San Jose, CA, USA).

### Dual-luciferase assay

The Promoter 2.0 Prediction Server was used to predict the possible binding sites of Mct-1 in the Yap promoter region. A sequence of about 1400 bp upstream of the whole Yap gene was identified and cloned into a pGL3-Basic luciferase reporter vector (Corues Biotechnology, Nanjing, China). A Dual-Lumi™ II Luciferase Reporter Gene Assay Kit (Beyotime Institute of Biotechnology, Haimen, China) was used to detect double luciferase activities. Cells were transfected with the luciferase reporter constructs containing the promoter and luciferase activities were measured after 48 h using the Dual-Luciferase Reporter Assay System (Promega Corporation, Madison, WI, USA).

### Tumor xenograft models

Female nude mice (BALB/C-nu/nu, 4–6-weeks old) were purchased from the Laboratory Animal Resources Center of Nanjing Medical University and randomly assigned to one of four groups (MHCC97H-shMct-1/shNC, Hep3B-Mct-1/vector). Each mouse was injected with 1 × 10^6^ cells in 100 μL of PBS. The tumor size and body weight were recorded every 3 days. The mice were sacrificed on post-inoculation day 30. The protocols of all animal experiments were approved by the Institutional Animal Care and Use Committee of Nanjing Medical University.

### Statistical analysis

The data are expressed as the mean ± standard error of experiments repeated at least three times. IBM SPSS Statistics for Windows, version 23.0. (IBM Corporation, Armonk, NY, USA), Adobe Photoshop CS6 (Adobe, Inc., San Jose, CA, USA), and GraphPad Prism 8.0 software (GraphPad Software, Inc., San Diego, CA, USA) were used to process the data. The Student’s *t*-test was used to identify statistical differences between two groups, while the chi-squared test was used to identify correlations between Mct-1 expression and clinic pathological factors. The overall survival (OS) and recurrence-free survival (RFS) rates were calculated by the Kaplan–Meier method, and differences were assessed with the log-rank test. Person correlation analysis was employed to analyze the correlation between the expression patterns of Mct-1 and Yap in liver cancer. A probability (*p*) value of <0.05 was considered statistically significant.

## Supplementary information

Figure S1

Figure S2

Supplementary figure legends

## Data Availability

All data generated or analyzed during this study are included in this published article.
