# Supplementary figures and images for: The oncogene Mct-1 promotes progression of hepatocellular carcinoma via enhancement of Yap-mediated cell proliferation
Source: Cell Death Discov. 2021 Mar 22;7:57. doi: 10.1038/s41420-021-00413-3 (PMC7985373; doi:10.1038/s41420-021-00413-3)

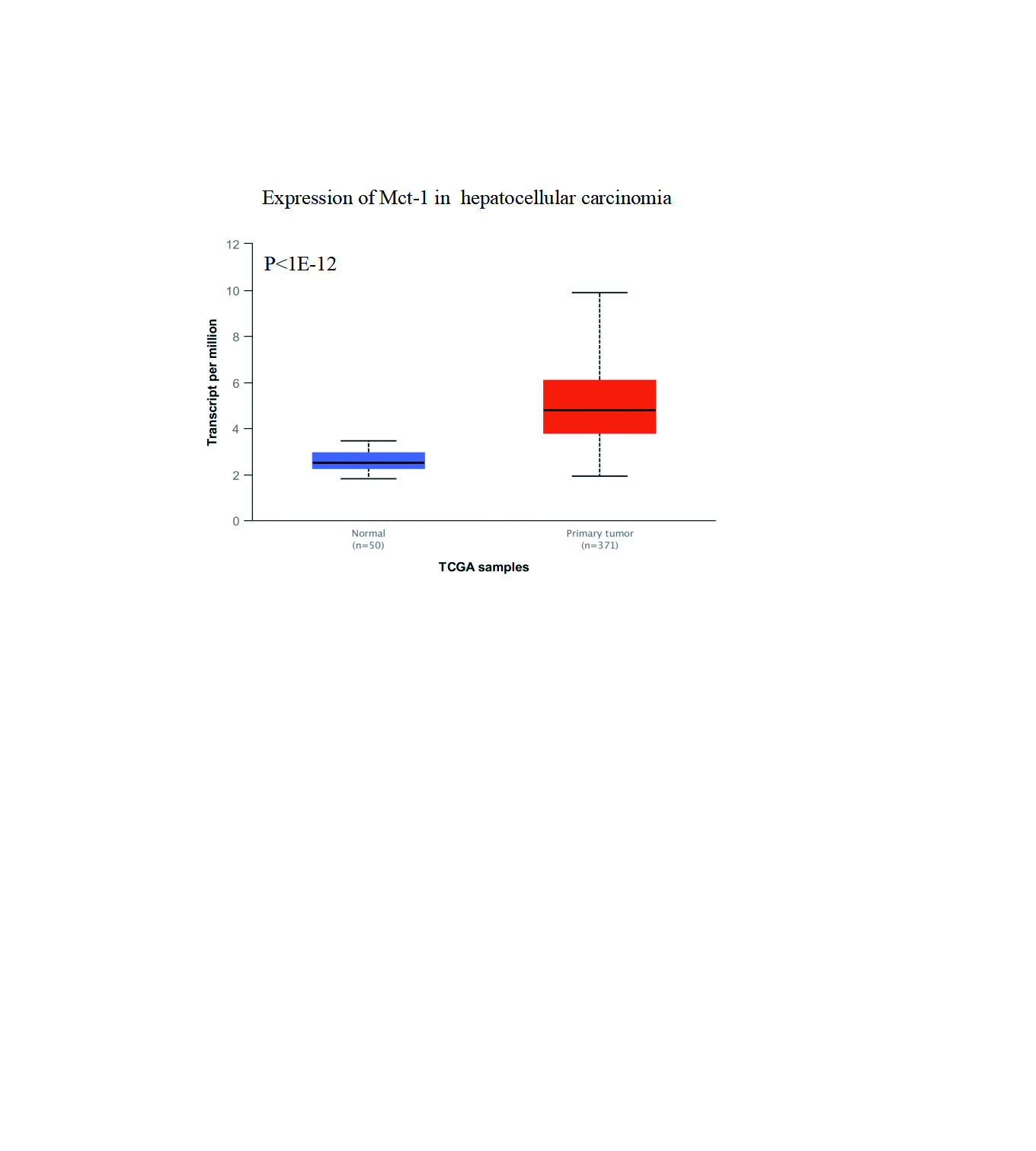

Supplement: Supplementary file 1 — Figure S1 [file 41420_2021_413_MOESM1_ESM.tif]

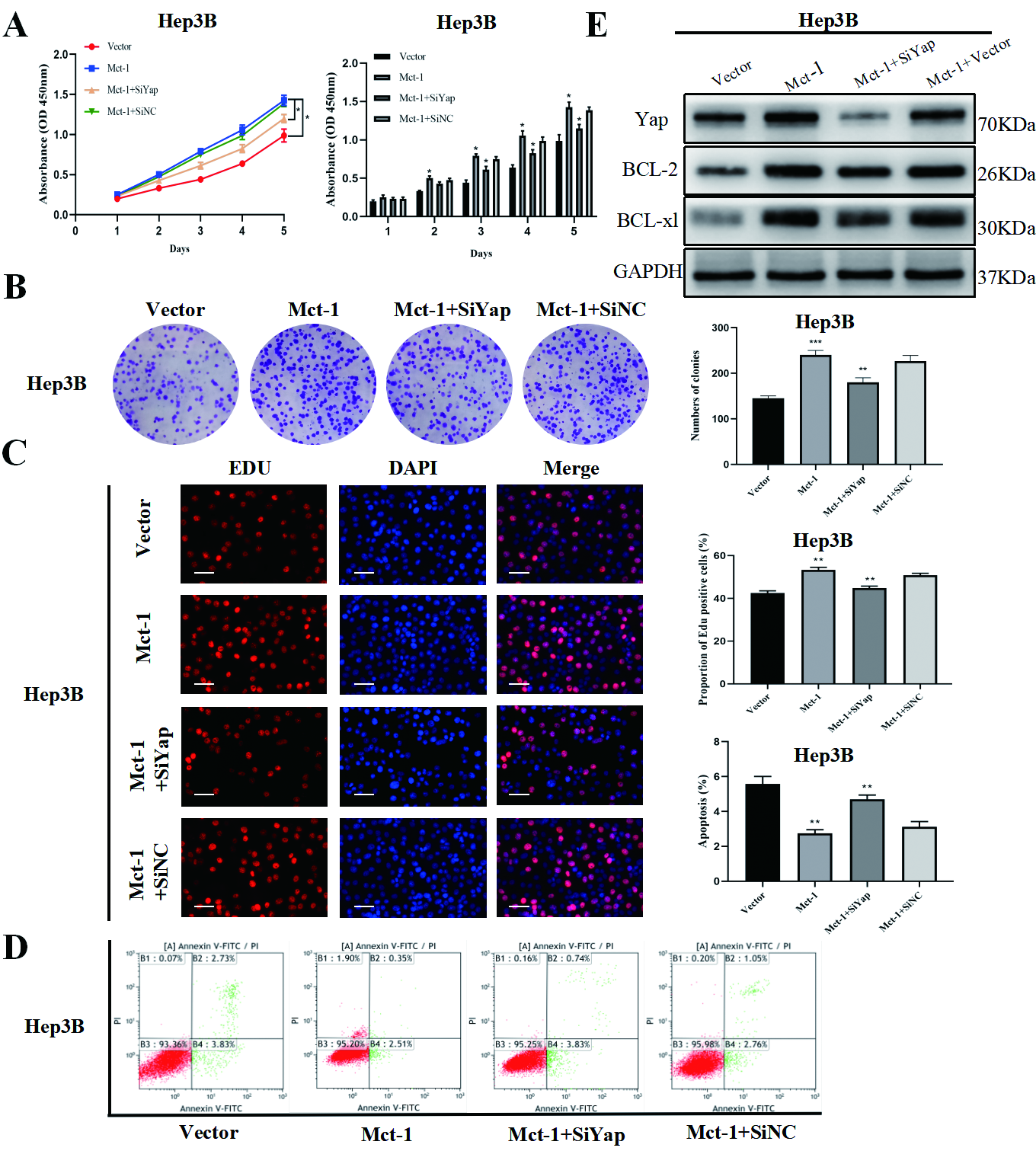

Supplement: Supplementary file 2 — Figure S2 [file 41420_2021_413_MOESM2_ESM.tif]
