# Supplementary material for: The oncogene Mct-1 promotes progression of hepatocellular carcinoma via enhancement of Yap-mediated cell proliferation
Source: Cell Death Discov. 2021 Mar 22;7:57. doi: 10.1038/s41420-021-00413-3 (PMC7985373; doi:10.1038/s41420-021-00413-3)
Supplement: Supplementary file 3 — Supplementary figure legends [file 41420_2021_413_MOESM3_ESM.docx]

**Fig S1** Mct-1 expression in 371 HCC tissues samples as compared to adjacent normal tissues (TCGA datasets).

**Fig S2** Yap expression is critical for Mct-1-mediated promotion of cell proliferation and inhibition of cell apoptosis. (**A–D**) CCK-8, colony formation, Edu, and cell apoptosis analyses of Mct-1-overexpression Hep-3B cells transfected with the Yap siRNA or siNC. Scale bar=50 µm. (**E**) Protein expression of Yap together with the anti-apoptotic proteins Bcl-2 and Bcl-xl in Hep 3B cells transfected with the Mct-1 overexpression lentivirus (Mct-1) and Yap siRNA. The data are presented as the mean ± SD of experiments performed in triplicate. **p* < 0.05, ***p* < 0.01, ****p* < 0.001.
